# Special Issue on Ophthalmic Optics and Visual Function

**DOI:** 10.3390/jcm11112966

**Published:** 2022-05-24

**Authors:** Kazuno Negishi

**Affiliations:** Department of Ophthalmology, Keio University School of Medicine, 35, Shinanomachi, Shinjuku-ku, Tokyo 160-8582, Japan; kazunonegishi@keio.jp

Exploring quality of vision is one of the most important issues in modern ophthalmology, and research into ophthalmic optics and visual function is essential for making progress in this field. Several factors affect quality of vision, and among them, refractive error/aberrations [1,2], accommodation [3], and tear film [4] are major.

People’s lifestyles have changed dramatically in recent decades, and a variety of digital devices, including personal computers and gadgets, are used extensively in daily life for social and professional purposes across all age groups. These changes have resulted in a range of ocular and visual symptoms [5].

Uncorrected/under-corrected refractive errors, aberrations, and presbyopia accelerate the multifaceted symptoms of the so-called digital computer syndrome, including eye strain, asthenopia, and other symptoms [5,6]. Dry eye may also accelerate the symptoms, because the tear film plays an important role as the first refractive ocular component, and the alterations in the tear film dynamics may cause vision-related and ocular surface-related symptoms [4].

The recent lifestyle change may also contribute to the increased prevalence of myopia because environmental factors are considered to be important for myopia progression [7,8,9].

This Special Issue of *JCM* on “Ophthalmic Optics and Visual Function” is a collection of articles that highlight innovative findings with the potential of enhancing diagnosis and monitoring ophthalmic conditions and treatments, especially of the anterior segment.

The issue includes 16 manuscripts: two original papers on refraction measurement, four on presbyopia diagnosis and treatment, two on myopia treatment, four on other topics, and one review paper and three original papers on myopia control.

Regarding presbyopia, Yang et al. evaluated the impact of myopia severity and the type of visual correction in presbyopia on vision-related quality of life (QOL) and reported that highly myopic presbyopes had a worse overall QOL and functionality, both with and without glasses, compared to presbyopes with low myopia, although progressive addition lens users had a better perception outcome than single-vision distance lens users in both groups [10]. Kubota et al. investigated the factors that cause presbyopia other than advanced age and reported that age and the difference between the maximal and minimal pupillary diameters were both significantly and independently related to accommodation amplitude and age under 44 years but not age 45 years and older [11]. Tsuneyoshi et al. reported that patients became aware of presbyopia in their late forties, although some had difficulty with near-vision-related tasks before becoming aware of presbyopia [12]. These studies suggest that proper intervention for presbyopia may improve the quality of vision and vision-related QOL.

Yotsukura et al. reported the prevalence of myopia in equatorial Brazil and suggested that the light environment, in addition to other confounding factors, affects the axial length and refractive errors [13]. Ishiko et al. reported the effect of educational pressure on myopia progression and reported that the progression rates and increased prevalence of high myopia were observed only during high-pressure education [14]. Tsai et al., who conducted a systematic review and meta-analysis with the latest evidence on the efficacy and safety of 0.01% atropine in myopic children, concluded that the drug had favorable efficacy and adequate safety for childhood myopia over a 1-year period [15]. Mori et al. conducted a randomized controlled trial on the effect of violet light-transmitting eyeglasses on axial elongation in myopic children and reported that the mean change in axial length in the violet light glasses group was significantly smaller than in the placebo glasses group when the time spent performing near work was less than 180 min and when the subjects were limited to those who had never used eyeglasses before this trial [16].

These reports support the relationship between environmental factors and myopia progression as previously reported and added new findings.

Other studies in this Special Issue are on the visual function related to cataract [17] and lacrimal passage intubation [18], clinical results and QOL related to surgeries [19,20,21], refractive measurement [22,23], and others [24,25].

As Guest Editor, I thank the reviewers for their professional comments and the JCM Editorial Office for their robust support. I believe the readers of this Special Issue will find the articles very useful.
